# Neural Substrates for the Motivational Regulation of Motor Recovery after Spinal-Cord Injury

**DOI:** 10.1371/journal.pone.0024854

**Published:** 2011-09-28

**Authors:** Yukio Nishimura, Hirotaka Onoe, Kayo Onoe, Yosuke Morichika, Hideo Tsukada, Tadashi Isa

**Affiliations:** 1 Department of Developmental Physiology, National Institute for Physiological Sciences, Okazaki, Japan; 2 Precursory Research for Embryonic Science and Technology, Japan Science and Technology Agency, Tokyo, Japan; 3 Core Research for Evolutional Science and Technology, Japan Science and Technology Agency, Kawaguchi, Japan; 4 Functional Probe Research Laboratory, RIKEN Center for Molecular Imaging Science, Kobe, Japan; 5 Molecular Dynamics Laboratory, RIKEN Center for Molecular Imaging Science, Kobe, Japan; 6 Central Research Laboratory, Hamamatsu Photonics, Hamamatsu, Japan; 7 Graduate University for Advanced Studies (SOKENDAI), Hayama, Japan; University of Alberta, Canada

## Abstract

It is believed that depression impedes and motivation enhances functional recovery after neuronal damage such as spinal-cord injury and stroke. However, the neuronal substrate underlying such psychological effects on functional recovery remains unclear. A longitudinal study of brain activation in the non-human primate model of partial spinal-cord injury using positron emission tomography (PET) revealed a contribution of the primary motor cortex (M1) to the recovery of finger dexterity through the rehabilitative training. Here, we show that activity of the ventral striatum, including the nucleus accumbens (NAc), which plays a critical role in processing of motivation, increased and its functional connectivity with M1 emerged and was progressively strengthened during the recovery. In addition, functional connectivities among M1, the ventral striatum and other structures belonging to neural circuits for processing motivation, such as the orbitofrontal cortex, anterior cingulate cortex and pedunculopontine tegmental nucleus were also strengthened during the recovery. These results give clues to the neuronal substrate for motivational regulation of motor learning required for functional recovery after spinal-cord injury.

## Introduction

Current basic researches on the neuro-rehabilitation focus mainly on neuronal plasticity and motor learning for functional restoration of affected limbs after neuronal damage such as spinal-cord injury and stroke. Depression is a common psychological problem after neuronal damage [1]–[4], and it has been shown to impede the recovery process [3]. Therefore, motivation is a key issue for enhancing the effect of rehabilitation therapy. However, the neuronal substrate underlying such psychological effects on functional recovery remains obscure.

**Figure 1:**
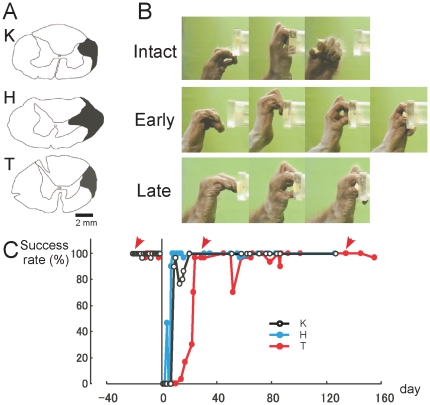
Recovery of finger dexterity after spinal-cord injury. (**A**) Extent of the l-CST lesion at the C4–C5 segment (indicated by black hatch) in three monkeys. (**B**) Representative video frames showing the recovery of finger dexterity in monkey T. Red arrows towards B indicate the corresponding day on the plot of recovery time course. Intact: Day −20, Early: Day 27, Late: Day 137. (**C**) Recovery time course of precision grip in three monkeys. A successful trial was defined as any trial that resulted in the successful precision grip with the index finger and thumb and removal of the food from the pin without dropping it. The graph in C was modified from our previous study [6] with permission.

**Figure 2:**
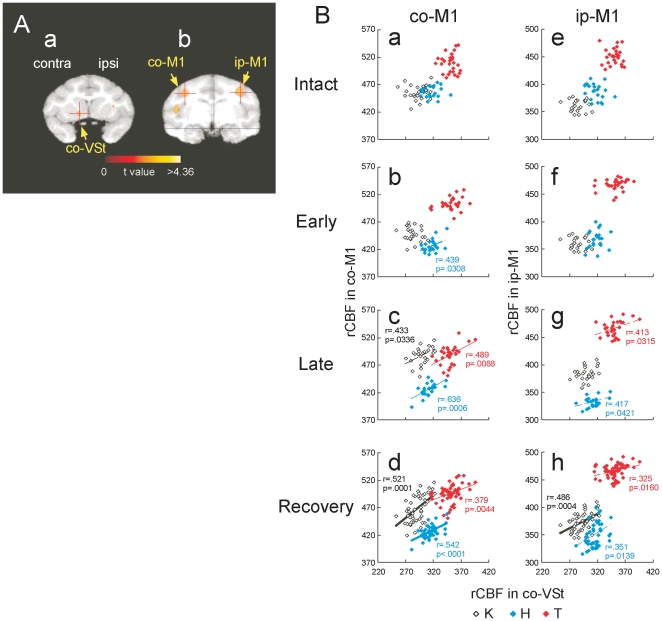
The correlation of rCBF between the ventral striatum (VSt) and the primary motor cortex (M1) before and after spinal-cord injury. (**A**) Region of interest (ROI) in the ventral striatum (**a**, co-VSt), contralesional primary motor cortex (left on **b**, co-M1) and ipsilesional primary cortex (right on **b**, ip-M1). Each ROIs were indicated by crossed point of the red lines. (**B**) The correlation of rCBF between the VSt and the M1 before and after the spinal-cord injury. Scatter plot diagrams show the correlation of rCBF between the VSt and the co-M1, and between the VSt and ip-M1 during intact (**a** and **e**), early recovery (1–2 months after lesion, **b** and **f**), late recovery (3–4 months after lesion; **c** and **g**) and recovery (including the data from both early and late recovery; **d** and **h**) stages. Individual data points indicate the data from individual sessions of the PET scan in each monkey that are shown with different symbols. Numerals beside the regression lines indicate Pearson's correlation coefficients (*r*) (dotted line: *P*<0.05, thin line: *P*<0.01, bold line: *P*<0.001) and *P*-values (*p*). The plots without the *r* and *p-value*, indicate no significant correlation (All the *r*- and *p*-values are shown in Table S1).

Recently, we showed the neuronal mechanisms of functional recovery in a non-human primate model of partial spinal cord injury (Fig. 1A) [5]–[9]. Advantages of the experiments on non-human primate models are, for instance, that the extent of the lesion can be well defined and that the recovery time course can be controlled. Furthermore, longitudinal and quantitative behavioral evaluation from pre-lesional state are possible which is usually unavailable in patient studies [7], [10]. Moreover, especially the macaque monkeys are indispensable model animals for this kind of research, because they are close enough to humans both in the structure of the brain and body architecture [11], [12]. Especially loss of the dexterous hand movements is one of the major causes of suffering for the patients with spinal cord injury and such movement repertories are equipped only in higher primate species including humans and macaque monkeys. The macaque monkeys were trained to reach for a small piece of food through a narrow vertical slit, and to grasp it between the pads of their index finger and thumb (Fig. 1B, see Video S1 for details). We performed a near-complete or complete lesion of the lateral corticospinal tract (l-CST) at the mid-cervical spinal segment, which impaired the direct cortico-motoneuronal connections while the indirect pathways from the motor cortex to motoneurons, mediated by brainstem or spinal cord interneurons whose axons were located ventrally to those of the l-CST, were mostly left intact (Fig. 1A). As described in the previous study, the precision grip was impaired immediately after the spinal-cord injury. However, the success rate of precision grip recovered almost completely within 4–5 weeks in all the 3 monkeys tested (Fig. 1B and C, see Video S1 for details). We performed PET scans during the preoperative, early (approximately 3 weeks to 1.5 month postoperatively) and late stages of recovery (approximately 3–4 months postoperatively) and measured the regional cerebral blood flow (rCBF) as an index of the neuronal activity. We found that activities of the bilateral M1 increased during the early recovery stage (Fig. 2Ab). During the later stage of recovery, the activity of the bilateral ventral premotor cortices (PMv) increased and the activated area of the contralesional M1 was expanded, while activation of the ipsilesional M1 was reduced. Moreover, the contribution of these cortical regions to recovery has been demonstrated by a reversible inactivation with the microinjection of muscimol. On the other hand, we also found that the activity of the contralesional ventral striatum (VSt), including the nucleus accumbens (NAc), increased during the recovery (Fig. 2Aa) [6], [7]. In this study, in order to explore the relationship between these motor cortices and motivation centers, we examined the functional connectivity of the VSt with other brain regions by the correlational analysis of rCBF [13], [14] using the same set of the PET data in our preceding study [6].

## Results

### Functional recovery of finger dexterity after l-CST lesion

As described in previous study [6], dexterous finger control was impaired immediately after the spinal-cord injury. The success rate of precision grip recovered to more than 80% of that before the lesion within 3 weeks in all the 3 monkeys. Then the success rate recovered almost completely within 4–5 weeks (Fig. 1A and B). The recovery time course of each animal was not uniform (Fig. 1C), although we intended the lesions to be as similar in size as possible (Fig. 1A). The timing of PET scans at each stage was determined by the recovery level in the behavioral test. We performed the PET scans when each monkey reached similar performance level. On the basis of recovery course of precision grip, we performed PET scanning during the following three stages: (i) preoperative stage, (ii) early recovery stage, and (iii) late recovery stage. PET scans during the early recovery stage were initiated when the success rate for retrieval reached 80%. PET scans during the late recovery stage were initiated when the success rate for retrieval consistently reached 100% at 3 months post-operation. In Monkey H, PET scans during the early recovery stage were conducted between postoperative days 15 to 29, and those during the late recovery stage were conducted between postoperative days 90 to 112. In Monkey K, PET scans during the early stage were conducted between postoperative days 22 to 41, and those during the late stage were conducted between postoperative days 92 to 115. In Monkey T, PET scans during the early stage were conducted between postoperative days 36 to 64, and those during the late stage were conducted between postoperative days 106 to 122.

### Functional connectivity between the ventral striatum and motor cortex emerged and was strengthened through the recovery course

Regions of interest (ROIs) for the contralesional ventral striatum (co-VSt, Fig. 2Aa), contralesional motor cortex (co-M1, Fig. 2Ab) and ipsilesional motor cortex (ip-M1, Fig. 2Ab), which included the activated foci revealed by SPM analysis, was determined by the results of the main effect of the functional recovery, which were reported in our previous study [6]. Scatter plot diagrams in Figure 2B show the correlations of signal intensity in the co-VSt with that in the co- (Fig. 2Ba–d) or ip- (Fig. 2Be–h) M1 in each monkey before and after the spinal-cord injury. A positive correlation between the co-VSt and both the co- and ip-M1 was not observed prior to the lesion (Fig. 2Ba and e), but emerged during the recovery, especially during the late recovery stage (Fig. 2Bb–d, Bf–h) in all the 3 monkeys. All the *r*- and *p*-values of correlation at the individual stages are indicated in Table S1.

### Functional connectivity of the ventral striatum with other motivation centers

**Figure 3:**
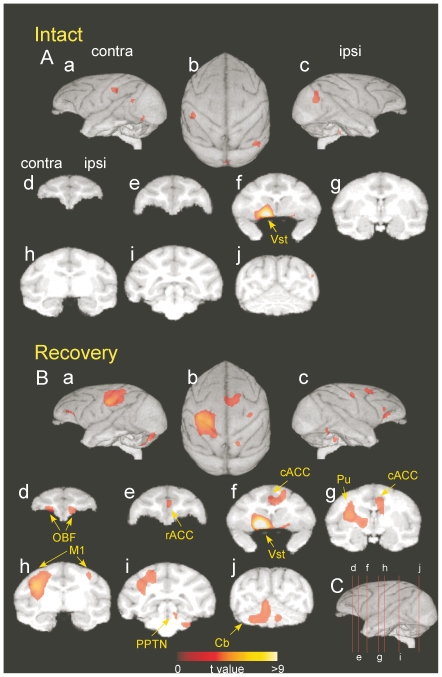
Strength of functional connectivity of individual brain regions with the contralesional ventral striatum before and after spinal-cord injury. (**A**) Preoperatively, (**B**) postoperatively. Average data obtained from the three monkeys. The correlations were calculated between the rCBF value of the ROI in the contralesional ventral striatum (co-VSt, see Fig. 2Aa) and that in other regions during the precision grip task. Brain areas that have significant positive correlation (*P*<0.01, uncorrected for multiple comparisons) are indicated on a three-dimensional reconstruction of a template brain MRI of macaque monkeys. The significance level is given in terms of t-values represented in a colored scale. (**A**) and (**B**) are the results during the intact and recovery stages (including the data from both early and late recovery), respectively. Views are from the contralesional hemisphere (**a**), top (**b**), ipsilesional hemisphere (**c**), and coronal sections (**d**–**j**). Lines drawn on the lateral views of the brain in **C** correspond to the levels of coronal sections of (**d**–**j**) in **A** and **B**.

Summary of multiple comparisons of the whole brain regions of the 3 monkeys are shown in Figure 3 and Table 1. During the recovery, significant positive correlations with the VSt (ROI in Fig. 2Aa) were also observed in bilateral orbitofrontal cortices (OBF, Fig. 3Bd), the rostral part of the anterior cingulate cortex (rACC, Fig. 3Be), the caudal part of the ACC (cACC, Fig. 3Bf and g), the ipsilesional pedunculopontine tegmental nucleus (PPTN, Fig. 3Bi) and the putamen (Pu, Fig. 3Bg), all of which have been described to be involved in the processing of motivation and emotion [15]–[28] (see also Table 1). Such correlation was not observed preoperatively, but, emerged during recovery (Fig. 3).

**Table 1:** Statistical analysis of correlation of the rCBF in the co-VSt with that in other brain regions during the recovery stage.

Brain region	Laterality	t-value	Location in Fig. 3B
OBF	contra	3.73	d
OBF	ipsi	3.42	d
rACC	ipsi	3.18	e
cACC	ipsi	3.37	f
Pu	contra	4.26	g
M1	contra	5.46	H
M1	ipsi	3.19	h
IPS	contra	4.31	i
PPTN	ipsi	3.06	i
Cb	ipsi	3.40	j
Cb	Contra	3.94	j

The level of the significance was set at P<0.001 (t>3.15). The t-values at the center of individual masses of activation (the locations are indicated with letters that correspond with those in Fig. 3Bd–j) are indicated.

**Figure 4:**
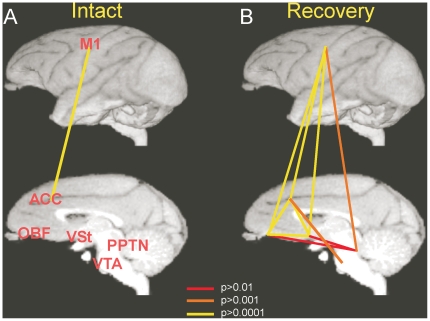
The functional connectivity among individual brain regions during the preoperative and postoperative stages. Strength of functional connectivity between two regions out of M1, VSt, ACC, OBF, PPTN and VTA are indicated by the color of individual lines connecting the two regions. (**A**): Intact, (**B**): Recovery stage. The colors of lines indicate the intensity of the connectivity represented by *p*-values of correlation.

To confirm whether these brain sites are mutually correlated, we calculated correlation of signal intensity within these brain sites. In figure 4, the significant level of correlation between two areas among M1, ACC, OBF, VSt, PPTN and Ventral Tegmental Area (VTA) in the intact state and during the recovery process is indicated with the color of the lines connecting the two areas. As clearly indicated, only the M1 and ACC showed positive correlation in the intact state (Figure S2Ab–c and S4Aa, Table S2 and S5 in intact). However, the correlation between the M1 and these motivation centers including the ACC, OBF, VSt, PPTN and VTA, and correlation within motivation centers were strengthened during the recovery process (Figure S1, S2, S3, S4, S5, S6, Table S2, S3, S4, S5, S6, S7). Thus, functional connectivity between the M1 and the motivation centers and mutual functional connection among the motivation centers were strengthened throughout the period of recovery process.

## Discussion

The present study has shown that the functional connectivity among the M1, the VSt and other structures belonging to the neural circuits for processing motivation emerged and were strengthened during recovery. The present study thus demonstrated plasticity in the functional connectivity of the motivational networks with the M1, which might be specifically correlated with the extent of functional recovery of finger dexterity after the spinal-cord injury.

The VSt, in particular the NAc, has been shown to be important in information processing for motivation and reward. Neuronal activity [21] and dopamine [22] release in the NAc have been reported to increase during expectation and experience of rewards. Patients with dysfunction of the NAc suffer from depression [24], and the NAc may act as a gateway to enhance or degrade processing of motivational information that controls emotional actions and behaviors [18], [19], [23], [24]. A recent clinical study demonstrated that deep brain stimulation to the NAc alleviated anhedonia during depression, and accompanied increases in activity of the fronto-striatal networks and limbic system [26]. Furthermore, repetitive transcranial magnetic stimulation (rTMS) to the prefrontal cortex has been used as a potential treatment in neuropsychiatric disorders such as depression [16], [17], [20]. In addition, the ACC has been shown to play an important role in effort-related decision-making [27], [28]. Thus, activity of fronto-striatal networks including the NAc may play a critical role in the control of motivation and its activation might be helpful to enhance motivational state during the functional recovery of finger dexterity after the spinal-cord injury.

It is important to examine how the “functional connectivity” of the motivational networks with the M1 observed in the present study emerged. One possibility that should be excluded is that it simply reflects the change in amount of reward. On this aspect, because the PET scans were performed after the success rate exceeded 80%, the amount of reward that the monkeys got during the sessions was constant both preoperatively and postoperatively, or slightly less only during the early recovery stage. Therefore, the correlation observed in this study was not likely to have resulted from pseudo-correlation between the increase in the reward amount and increased activity of M1, both of which might be parallel the recovery of success rate.

On the other hand, task difficulty would be increased after spinal cord injury, which needs higher level of motivation to achieve the demanding task that requires activation of mesolimbic system. Task difficulty might also require higher level of M1 activation, that might lead to emergence of the correlation between the two areas. We assume this may not be the sole explanation of the present result, because correlation became higher at the later stage of recovery when performance of the task became easier, than the early phase, when the performing the task was difficult. Thus, at this stage, it is difficult to make clear explanation on how the correlation between the VSt and M1 emerged during the recovery from the spinal-cord injury. Further experiments are needed, such as recording of neural activity from VSt, and testing the effects of activation/inactivation of the VSt neural activity on the M1 activity and functional recovery of the hand dexterity. However, it might be suggested that functional recovery of finger dexterity requires skill learning and may require higher level of M1 activity, and increased synaptic efficacy in the pathways between M1 and motivation centers through the NAc might underlie such reorganization of the neural circuits involving the M1. Then a question may arise on how the M1 and NAc are connected with each other. By using transsynaptic labeling technique, it has been shown that the NAc provides strong multisynaptic projections to all the body-part representations in the M1 through the substantia innominata [29], which is also a part of the VSt. rTMS on the M1 can evoke the release of dopamine in the VSt, including the NAc, in anesthetized monkeys [30]. Thus, the M1 and NAc have oligosynaptic mutual interconnections.

It has been reported that the dopaminergic system is essential for reinforcement learning [31]–[33]. Currently, skill learning and reinforcement learning tend to be discussed in different contexts. Our results might have identified the neural substrates that bridge the reinforcement learning to motor learning for functional recovery after the neuronal damage. The present findings may promote the development of psychological therapies that enhance the efficacy and quality of rehabilitation.

## Methods

### Subjects

Three monkeys [two *Macaca mulatta* (Monkey T: male 8.1 kg, Monkey K: male 6.5 kg) and a *Macaca fuscata* (Monkey H: female 6.7 kg)] were used in the present study. The data of the present study were obtained for our previous studies [6], [29] and re-analyzed with a different method for the present study. The experiments were approved by the animal experimental committee of the National Institute of Natural Sciences (Approved No.: A15-85-58) and were performed in accordance with the NIH Guidelines for the Care and Use of Laboratory. Monkeys were monitored closely and animal welfare was assessed on a daily basis, and if necessary several times a day. This includes veterinary examinations to make sure animals are not suffering. If animals experience pain they receive pain medications. If pain cannot be relieved, or if veterinary examination reveals signs of suffering that cannot be relieved by analgesics, antiemetics, or antibiotic therapy, animals are euthanized. The details of animal welfare and steps taken to ameliorate suffering were in accordance with the recommendations of the Weatherall report, “The use of non-human primates in research”.

### Spinal Cord Injury

In order to obtain a partial spinal-cord injury, the l-CST located in the dorsolateral funiculus of the spinal cord was transected under general deep anesthesia [5]–[9]. Briefly, the border between the C4 and C5 segments was exposed by laminectomy of the C3 and C4 vertebrae, and a transverse opening was made in the dura. The CST lesion was made under a surgical microscope. First, a small opening in the pia mater was made at the lateral convexity of the spinal cord. A horizontal strip in a mediolateral direction relative to the lateral funiculus was made by inserting a minute L-shape hook that could not be inserted the gray matter. Next, the dorsal part of the lateral funiculus was transected using forceps from the dorsal root entry zone ventrally to the level of the horizontal strip of the lesion described above. Finally, the lesion was extended ventrally to the most lateral part of the lateral funiculus. The opened dura mater was closed, and skin and back muscles were sutured with nylon or silk strings.

### Behavioral test

As described in a previous report [5]–[9], in order to assess the capacity of dexterous finger movements before and after spinal cord injury, the monkeys were trained to be seated on a monkey chair and to reach, grasp and retrieve a small piece of sweet potato or carrot (about 7 mm cubic) through a narrow vertical slit using both the index finger and thumb of the forelimb (Fig. 1B, see also Video S1). The food piece was positioned in the center of a vertical slit located at the height of the monkey's shoulder and at a sagittal distance of 15 cm. Each experiment (approximately 30 min every third day) consisted of 100 trials. In each trial, the animal reached for the food piece from a fixed position. A high-speed digital video camera (33 frames/s at a shutter speed of 1/1000 of a second) was used to record the reach-retrieval sequences from a lateral view. A successful trial was defined as any trial that resulted in the successful precision grip with the index finger and thumb, and removal of the food from the pin without dropping it.

### PET experiments

A series of PET scans was conducted during the intact and recovery stages as descried elsewhere [6], [34] (see also text in RESULTS). Briefly, thirty-one slices of 3.6 mm intervals were collected simultaneously by the PET scanner. After the delivery of a bolus of [^15^O]H_2_O via a cannula placed into the sural vein, the scan was initiated automatically when the radioactivity in the brain reached greater than 30 kcps. The monkey was allowed to begin the behavioral task (20 trials) at about 10 s before the start of the PET scan. During the scan, the monkeys performed a series of reach-grip-retrieve-eat movements every 5 s. PET data were collected for 80 s (one 40 s frame followed by four 10 s frames). Video recording of the scanning session was performed, and if the monkey did not start from the fixed starting point or did not start reaching immediately (within less than 15 frames = 500 ms) after presentation of the food or did not move the hand directly to the food piece, the data from the session was excluded from analysis. Twenty scans were conducted in Monkey H and K in both tasks for every stage, and 24 scans were conducted in Monkey T.

### Data Analysis

The reconstruction was performed on projection data, after which images were corrected for attenuation using a transmission scan with a 4.0 mm Hanning filter. Reconstructed brain images (voxel size, 1.2×1.2×3.6 mm), which were scalped and smoothed with a 4.0 mm FWHM isotropic kernel, were processed using statistical analysis of the parametric mapping (SPM99) software. For the inter-subject analysis, brain shapes of individual monkeys were morphologically normalized to a pseudo-brain template MRI, which was constructed by averaging 8 brain MRI images from young adult male macaque monkeys using a [^18^F]FDG-PET image of each subject. PET images obtained from the scan sessions that satisfied the behavioral criteria were summated for their first 60 s epochs, and were used for statistical analysis by parametric mapping (SPM99) software (see Text S1). Regions of interest (ROIs) for the co-VSt, which included the activated foci revealed by SPM analysis, was determined by the results of the main effect of the functional recovery (Fig. 2Ab), which was reported in our previous study [6]. The correlations were calculated between rCBF values in the VSt on the contralesional hemisphere and in other regions during the precision grip task. Additional ROIs for the neuronal substrate for motivation such as the rostral and caudal part of the anterior cingulated cortex (rACC Fig. 3Be and cACC Fig. 3Bf), orbitofrontal cortex (OBF, Fig. 3Bd on contralesional side), and pedunculopontine tegmental nucleus (PPTN, Fig. 3Bi on ipsilesional mid-brain) were determined by the results of correlation analysis with rCBF on the VSt during the precision grip task at the recovery stage. Additional ROI for the motor center such as the primary motor cortex (M1, Fig. 3Bh on contralesional side) were also determined by the results of correlation analysis with rCBF on the VSt during the precision grip task at the recovery stage. The rCBF values of 9 voxels around the center voxel on each activation spot during each scan session were used for correlation analysis. The statistical threshold was set at *P*<0.01, uncorrected (*t*>2.38 for intact, early and late stage, *t*>2.35 for recovery stage).

### Histological assessment of lesion completeness

At the end of the experiments, the monkeys were deeply anesthetized with an overdose of sodium pentobarbital (50–75 mg/kg, i.v.) and perfused transcardially with 0.1 M phosphate-buffered saline (PBS, pH 7.3), followed by 4% paraformaldehyde in 0.1 M PBS (pH 7.3) and, finally, the same fresh PBS containing 10%, 20% and then 30% sucrose. The spinal cords were removed from the bone immediately, saturated with 30% sucrose in 0.1 M PBS (pH 7.3), then cut serially into 50 µm thick coronal sections on the freezing microtome. The sections processed for Klüver-Barrera staining or Nissl-staining with 1% Cresyl Violet were used for assessing the lesion extent.

## Supporting Information

Video S1 **Recovery time course of finger dexterity after spinal cord injury.**
(WMV)

Figure S1 **Strength of functional connectivity with the co-VSt.** Strength of functional connectivity derived from correlations between the rCBF value in the co-VSt and that in other regions during the precision grip task were calculated. The data were obtained from 3 monkeys. Brain areas with a significant positive correlated rCBF (P<0.01, uncorrected for multiple comparisons) are superimposed on a three-dimensional reconstruction of a template brain MRI of macaque monkeys that was made by our group. The significance level is given in terms of t values represented on a colored scale. (A), (B) (C) and (D) are results during the intact, early and late stages of recovery, and all recovery stages (including the data from both the early and late recovery stages), respectively. (a) to (g), coronal sections. (C) shows lateral views of the brain. Lines (d) to (f) in (C) indicate the levels of coronal sections of (a) to (g), respectively.
(TIF)

Figure S2 **Strength of functional connectivity with the co-M1.** The same arrangement as Figure S1.
(TIF)

Figure S3 **Strength of functional connectivity with the co-OBF.** The same arrangement as Figure S1.
(TIF)

Figure S4 **Strength of functional connectivity with the rACC.** The same arrangement as Figure S1.
(TIF)

Figure S5 **Strength of functional connectivity with the cACC.** The same arrangement as Figure S1.
(TIF)

Figure S6 **Strength of functional connectivity with the ip-PPTN.** The same arrangement as Figure S1.
(TIF)

Table S1 **Statistical analysis of correlation of the rCBF between the co-VSt and the primary motor cortex at different stages before and after the spinal-cord injury.** The values indicate Pearson's correlation coefficients (r) and p-values. r and p-values correspond with each scatter plot in figure 2B in main text. “n.s.” indicates no significant. co-M1: contralateral M1, ip-M1: ipsilateral M1.
(DOCX)

Table S2 **Statistical analysis of correlation of the rCBF in the co-VSt with that in other brain regions during the intact, early, late stage of recovery and recovery stage.** The level of the coefficients was set at P<0.01 (t = 2.38 for the intact, early, late stage of recovery, t = 2.35 for recovery stages). t-values at the center of individual masses of activation are indicated.
(DOCX)

Table S3 **Statistical analysis of correlation of the rCBF in the co-M1 with that in other brain regions during the intact, early, late stage of recovery and recovery stage.** The same arrangement as Table S2.
(DOCX)

Table S4 **Statistical analysis of correlation of the rCBF in the co-OBF with that in other brain regions during the intact, early, late stage of recovery and recovery stage.** The same arrangement as Table S2.
(DOCX)

Table S5 **Statistical analysis of correlation of the rCBF in the rACC with that in other brain regions during the intact, early, late stage of recovery and recovery stage.** The same arrangement as Table S2.
(DOCX)

Table S6 **Statistical analysis of correlation of the rCBF in the cACC with that in other brain regions during the intact, early, late stage of recovery and recovery stage.** The same arrangement as Table S2.
(DOCX)

Table S7 **Statistical analysis of correlation of the rCBF in the ip-PPTN with that in other brain regions during the intact, early, late stage of recovery and recovery stage.** The same arrangement as Table S2.
(DOCX)

Text S1 **Supporting information for Methods.**
(DOCX)
