# Supplementary figures and images for: Neural Substrates for the Motivational Regulation of Motor Recovery after Spinal-Cord Injury
Source: PLoS One. 2011 Sep 28;6(9):e24854. doi: 10.1371/journal.pone.0024854 (PMC3182173; doi:10.1371/journal.pone.0024854)

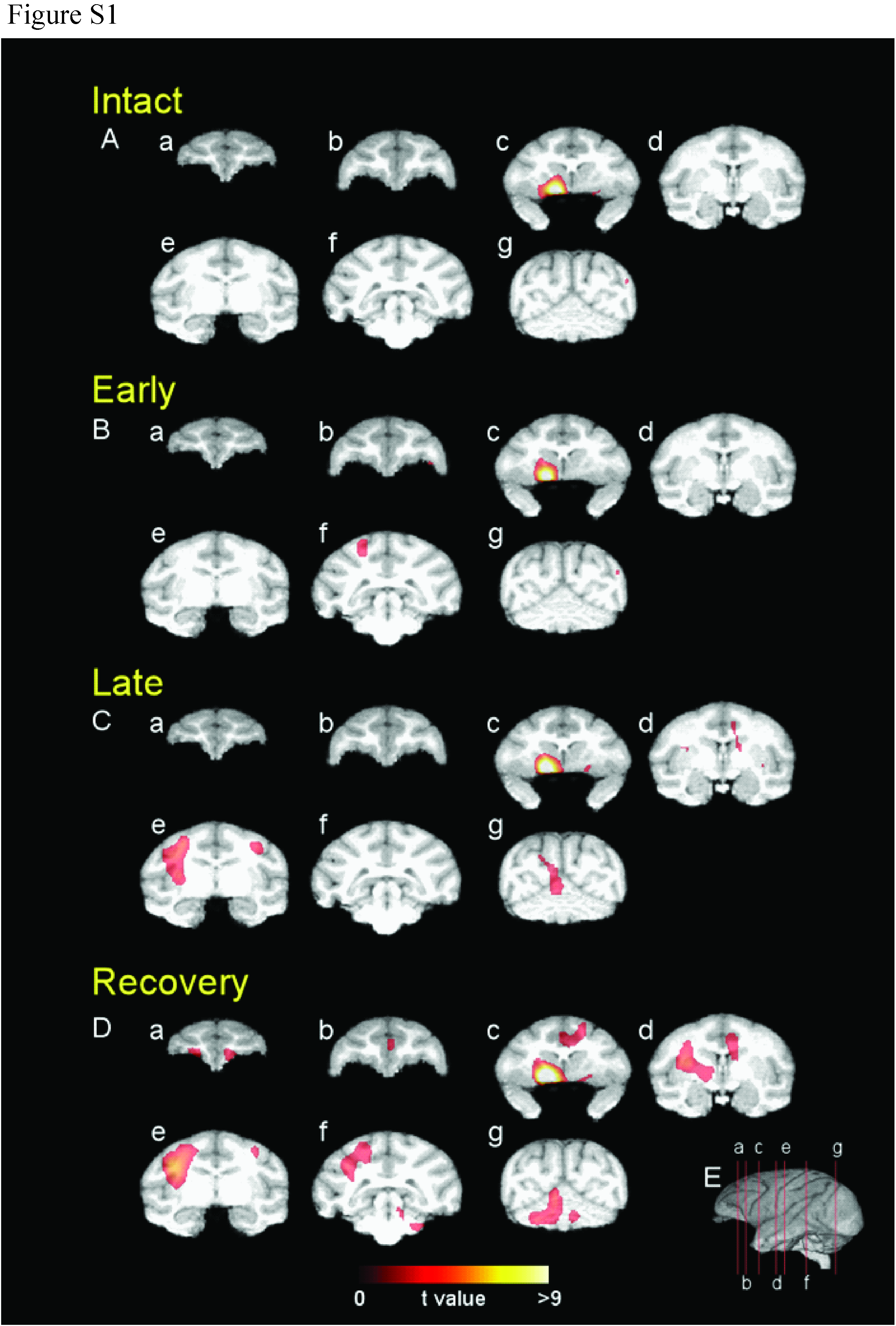

Supplement: Figure S1 — Strength of functional connectivity with the co-VSt. Strength of functional connectivity derived from correlations between the rCBF value in the co-VSt and that in other regions during the precision grip task were calculated. The data were obtained from 3 monkeys. Brain areas with a significant positive correlated rCBF (P<0.01, uncorrected for multiple comparisons) are superimposed on a three-dimensional reconstruction of a template brain MRI of macaque monkeys that was made by our group. The significance level is given in terms of t values represented on a colored scale. (A), (B) (C) and (D) are results during the intact, early and late stages of recovery, and all recovery stages (including the data from both the early and late recovery stages), respectively. (a) to (g), coronal sections. (C) shows lateral views of the brain. Lines (d) to (f) in (C) indicate the levels of coronal sections of (a) to (g), respectively. (TIF) [file pone.0024854.s002.tif]

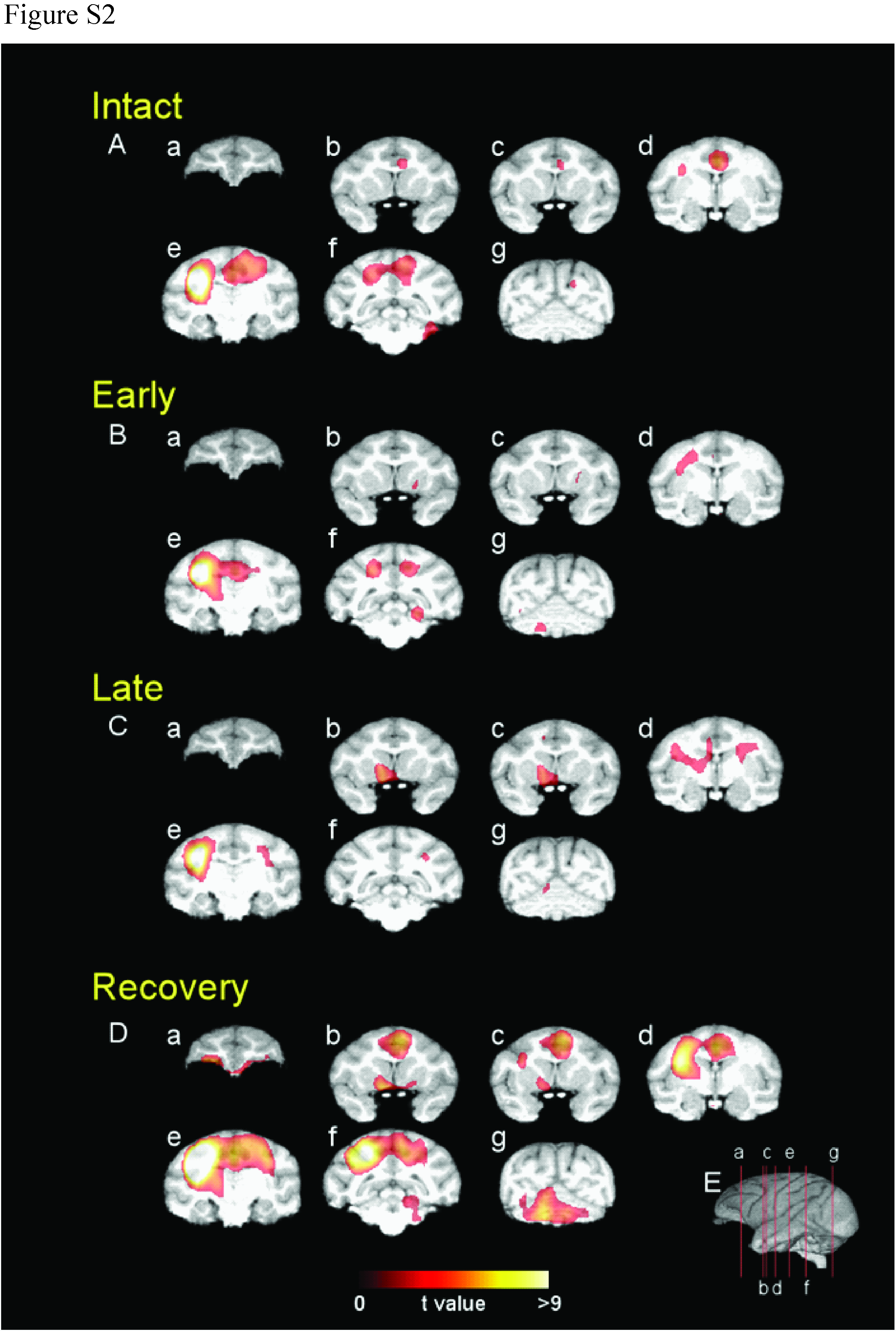

Supplement: Figure S2 — Strength of functional connectivity with the co-M1. The same arrangement as Figure S1. (TIF) [file pone.0024854.s003.tif]

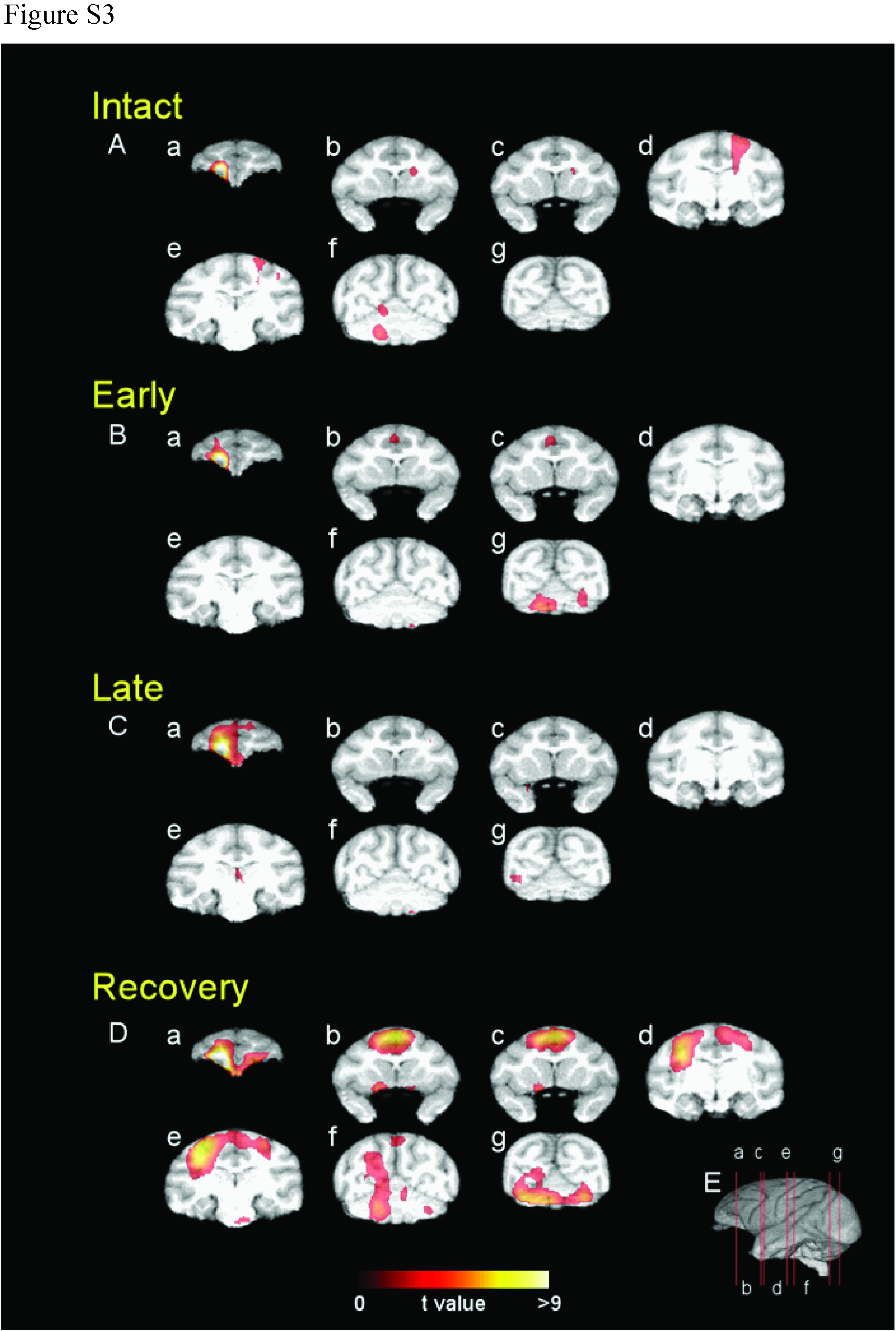

Supplement: Figure S3 — Strength of functional connectivity with the co-OBF. The same arrangement as Figure S1. (TIF) [file pone.0024854.s004.tif]

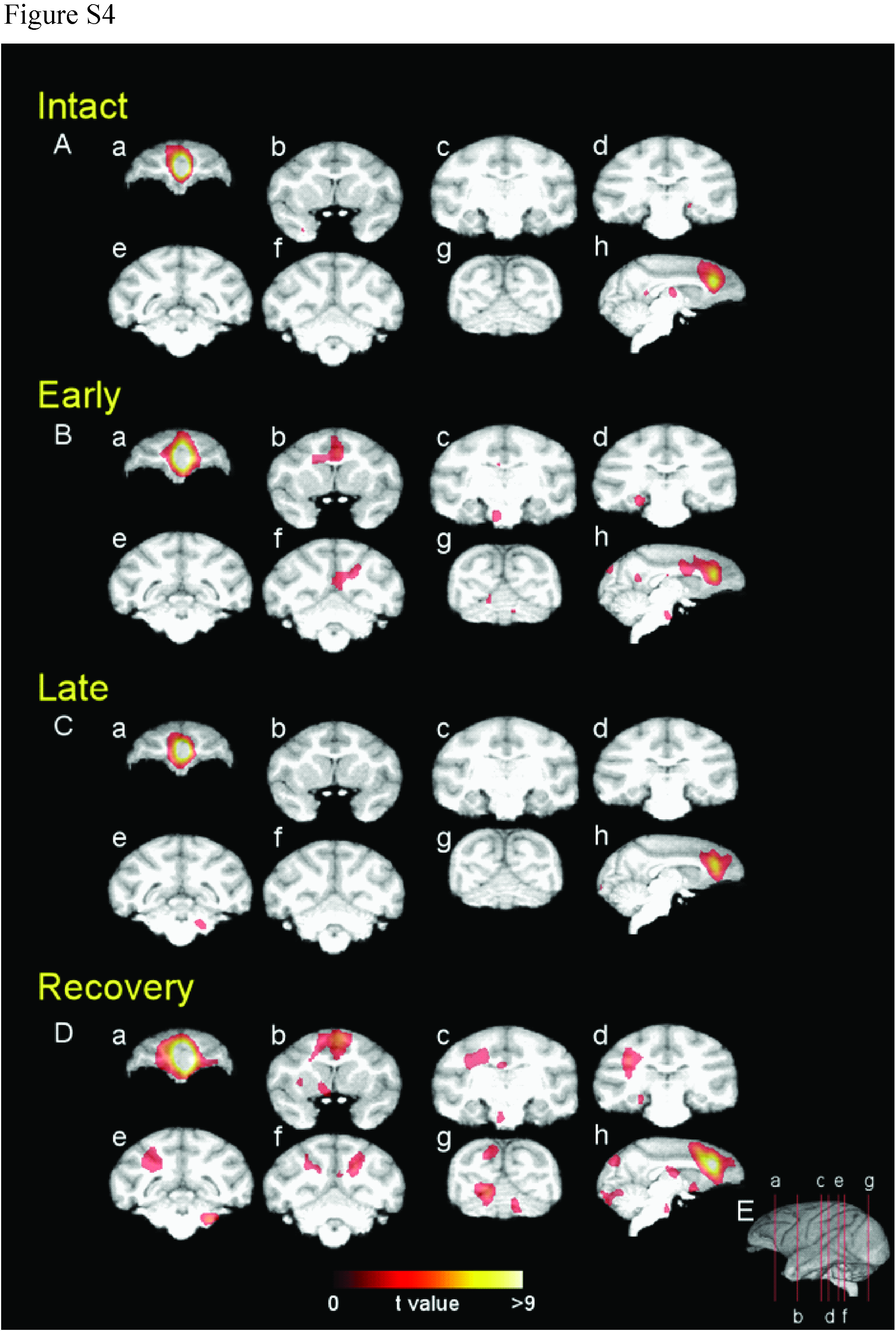

Supplement: Figure S4 — Strength of functional connectivity with the rACC. The same arrangement as Figure S1. (TIF) [file pone.0024854.s005.tif]

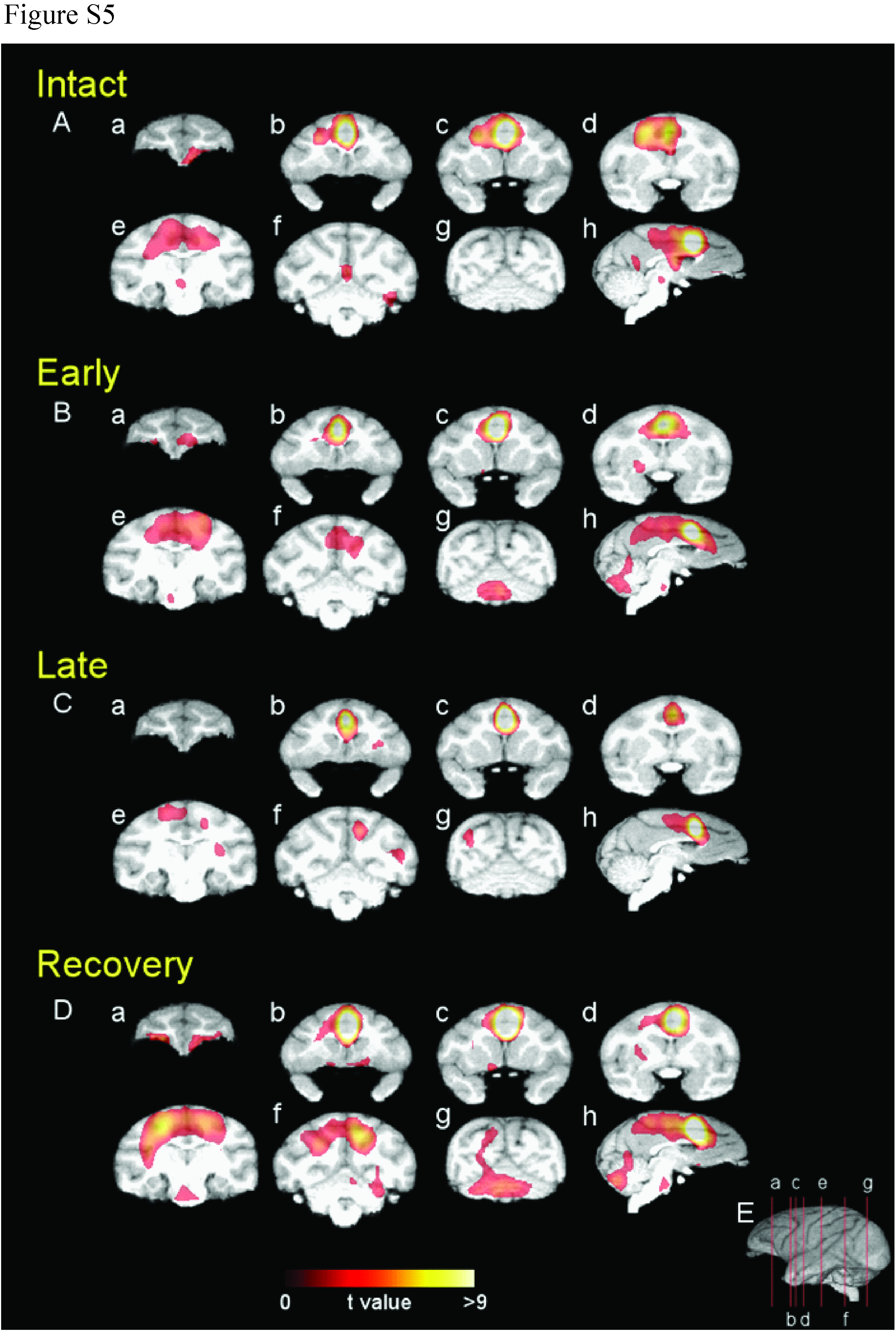

Supplement: Figure S5 — Strength of functional connectivity with the cACC. The same arrangement as Figure S1. (TIF) [file pone.0024854.s006.tif]

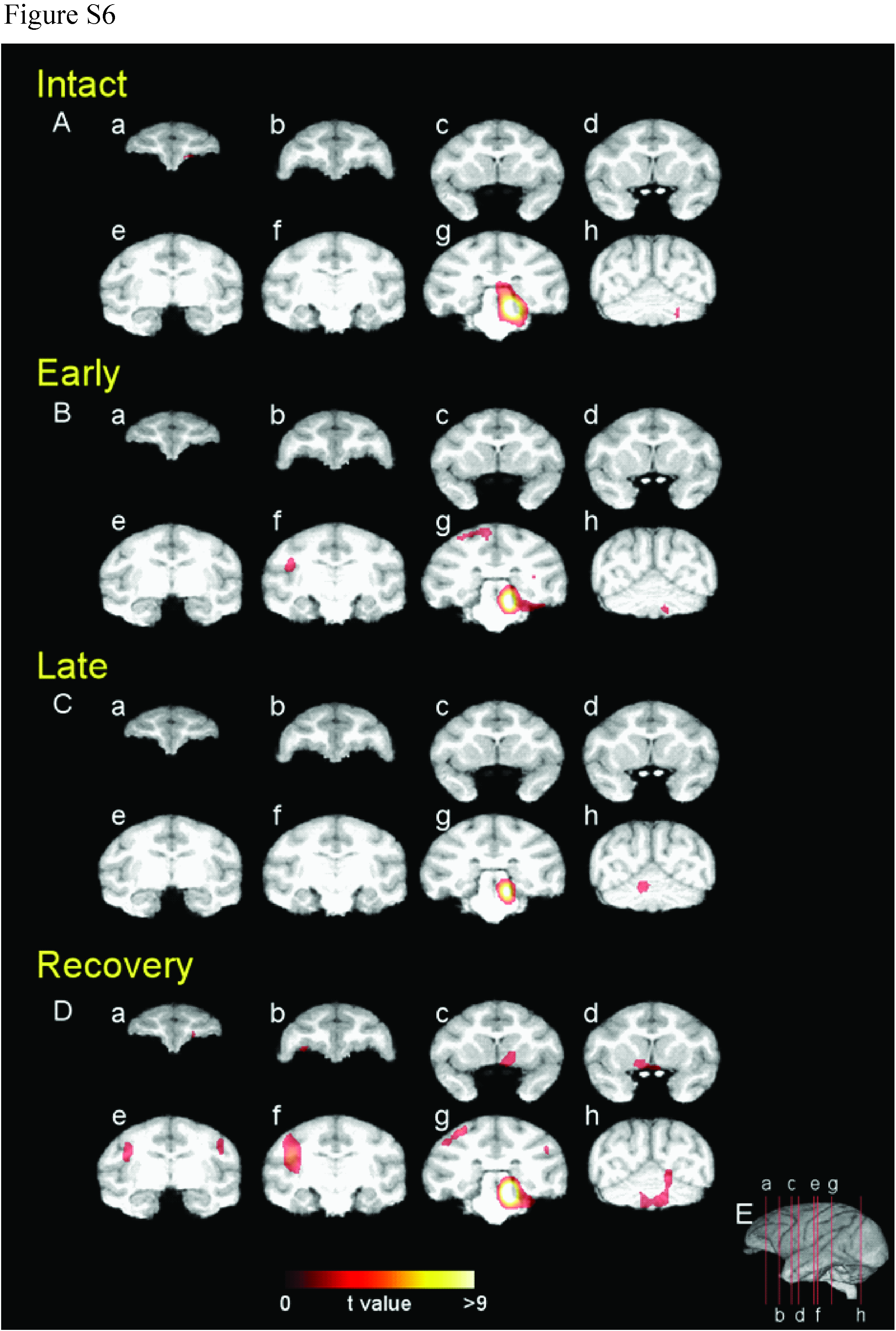

Supplement: Figure S6 — Strength of functional connectivity with the ip-PPTN. The same arrangement as Figure S1. (TIF) [file pone.0024854.s007.tif]
