# Supplementary material for: Neural Substrates for the Motivational Regulation of Motor Recovery after Spinal-Cord Injury
Source: PLoS One. 2011 Sep 28;6(9):e24854. doi: 10.1371/journal.pone.0024854 (PMC3182173; doi:10.1371/journal.pone.0024854)
Supplement: Table S1 — Statistical analysis of correlation of the rCBF between the co-VSt and the primary motor cortex at different stages before and after the spinal-cord injury. The values indicate Pearson's correlation coefficients (r) and p-values. r and p-values correspond with each scatter plot in figure 2B in main text. “n.s.” indicates no significant. co-M1: contralateral M1, ip-M1: ipsilateral M1. (DOCX) [file pone.0024854.s008.docx]

**Table S1**:

|  | co-M1 | ip-M1 |
| --- | --- | --- |
| **Monkey K**  Pre  Early  Late  Recovery | r= 0.181, p=0.4009, *n.s*  r=-0.154, p=0.4768, *n.s.*  r= 0.433, p=0.0336  r= 0.521, p<0.0001 | r=-0.066, p=0.7617, *n.s.*  r=-0.074, p=0.7389, *n.s.*  r= 0.367, p=0.0778, *n.s.*  r= 0.486, p=0.0004 |
| **Monkey H**  Pre  Early  Late  Recovery | r= 0.027, p=0.9032, *n.s.*  r= 0.439, p=0.0308  r= 0.636, p=0.0006  r= 0.542, p<0.0001 | r=-0.233, p=0.2770, *n.s.*  r= 0.223, p=0.2977, *n.s.*  r= 0.417, p=0.0421  r= 0.351, p=0.0139 |
| **Monkey T**  Pre  Early  Late  Recovery | r=-0.023, p=0.9114, *n.s.*  r=-0.307, p=0.1199, *n.s.*  r= 0.489, p=0.0088  r= 0.379, p<0.0044 | r=-0.041, p=0.8405, *n.s.*  r= 0.221, p=0.2708, n.s.  r= 0.413, p=0.0315  r= 0.325, p=0.0160 |
