# Supplementary material for: Neural Substrates for the Motivational Regulation of Motor Recovery after Spinal-Cord Injury
Source: PLoS One. 2011 Sep 28;6(9):e24854. doi: 10.1371/journal.pone.0024854 (PMC3182173; doi:10.1371/journal.pone.0024854)
Supplement: Table S2 — Statistical analysis of correlation of the rCBF in the co-VSt with that in other brain regions during the intact, early, late stage of recovery and recovery stage. The level of the coefficients was set at P<0.01 (t = 2.38 for the intact, early, late stage of recovery, t = 2.35 for recovery stages). t-values at the center of individual masses of activation are indicated. (DOCX) [file pone.0024854.s009.docx]

**Table S2**:

| Brain region | Laterality | t value |
| --- | --- | --- |
| **Intact**  Ca  Pu  Amygdala  AIP  MT  Cb  V2 or V4  V1 | Contra  Ipsi  Ipsi  Contra  Contra  Ipsi  Ipsi  Mid | 3.15  2.93  2.77  2.80  2.96  3.62  2.84  3.09 |
| **Early** |  |  |
| OBF  46  OBF  Cau  rACC  PCC  Insular  cACC  PMd  S1  Cb  Cb Vermis  Late  OBF  SMA  Pu  Pu  Ca  M1  M1  Insular  cACC  TPO  Cb  V6  V1  Recovery  OBF  OBF  ACC  VSt  PMd  Pu  PMv  Ca  M1  M1  Insular  IPS  PPTN  Cb  VIP  VIP  Cb  Cb  V6 | Ipsi  Contra  Contra  Contra  Contra  Ipsi  Contra  Ipsi  Ipsi  Contra  Contra  Mid  Ipsi  Ipsi  Contra  Ipsi  Ipsi  Contra  Ipsi  Contra  Mid  Ipsi  Contra  Contra  Contra  Contra  Ipsi  Ipsi  Ipsi  Ipsi  Contra  Contra  Ipsi  Contra  Contra  Contra  Contra  Ipsi  Ipsi  Ipsi  Contra  Contra  Contra  Contra | 2.52  2.84  3.69  2.87  2.55  3.49  2.90  2.61  2.52  3.25  2.84  2.61  2.87  3.19  2.43  2.74  2.96  3.94  3.53  4.36  2.99  3.18  3.65  2.58  2.77  3.73  3.42  3.18  2.84  3.38  4.26  2.74  3.12  5.46  3.19  4.31  4.31  3.06  3.40  3.13  2.49  3.94  3.15  2.46 |
