# Supplementary material for: Neural Substrates for the Motivational Regulation of Motor Recovery after Spinal-Cord Injury
Source: PLoS One. 2011 Sep 28;6(9):e24854. doi: 10.1371/journal.pone.0024854 (PMC3182173; doi:10.1371/journal.pone.0024854)
Supplement: Table S3 — Statistical analysis of correlation of the rCBF in the co-M1 with that in other brain regions during the intact, early, late stage of recovery and recovery stage. The same arrangement as Table S2. (DOCX) [file pone.0024854.s010.docx]

**Table S3**:

| Brain region | Laterality | t value |
| --- | --- | --- |
| Intact  rACC  cACC  Pu  Pu  M1  IPS  Cb  V6  Early  OBF  Pu  PMv  ACC  Insular  IPS  IPS  Cb  Cb  V2  Late  46v  VSt  VSt  ACC  PMv  M1  Insular  Pu  Insular  VIP  VIP  Cb  V1  Recovery  OBF  OBF  OBF  rACC  cACC  VSt  VSt  PMv  Pu  Insular  PCC  M1  Insular  Thalamus  IPS  IPS  PPTN  Cb  Cb  Cb  V1 | Ipsi  Mid  Contra  Ipsi  Ipsi  Contra  Ipsi  Ipsi  Ipsi  Ipsi  Contra  Mid  Contra  Contra  Ipsi  Ipsi  Contra  Contra  Contra  Contra  Contra  Contra  Contra  Ipsi  Contra  Contra  Ipsi  Contra  Ipsi  Contra  Contra  Contra  Mid  Ipsi  Mid  Mid  Contra  Ipsi  Contra  Contra  Contra  Mid  Ipsi  Ipsi  Contra  Contra  Ipsi  Ipsi  Ipsi  Contra  Ipsi  Contra | 3.62  6.54  8.07  4.60  4.41  5.35  3.07  2.99  2.84  2.91  2.99  4.09  5.43  4.88  3.23  3.54  3.54  2.68  2.68  3.47  4.72  3.54  2.91  3.47  8.74  5.91  2.99  4.80  2.99  3.62  2.52  5.42  3.94  3.56  3.81  5.45  6.23  4.41  5.23  8.97  19.6  5.98  5.51  3.23  4.31  14.3  4.10  3.37  4.10  6.30  4.72  2.76 |
