# Supplementary material for: Neural Substrates for the Motivational Regulation of Motor Recovery after Spinal-Cord Injury
Source: PLoS One. 2011 Sep 28;6(9):e24854. doi: 10.1371/journal.pone.0024854 (PMC3182173; doi:10.1371/journal.pone.0024854)
Supplement: Table S4 — Statistical analysis of correlation of the rCBF in the co-OBF with that in other brain regions during the intact, early, late stage of recovery and recovery stage. The same arrangement as Table S2. (DOCX) [file pone.0024854.s011.docx]

**Table S4**:

| Brain region | Laterality | t value |
| --- | --- | --- |
| Intact  Ca  M1  Cb  Cb  Early  46  rACC  PMd  cACC  Cb  Cb  Late  46v  rACC  46v  Insular  PN  Cb  V1  Recovery  OBF  46  ACC  cACC  VSt  PMd  M1  M1  IPS  IPS  Cb  Cb  PN | Ipsi  Ipsi  Contra  Ipsi  Contra  Mid  Ipsi  Ipsi  Ipsi  Contra  Contra  Contra  Ipsi  Ipsi  Contra  Contra  Contra  Ipsi  Contra  Contra  Mid  Contra  Contra  Contra  Ipsi  Contra  Ipsi  Contra  Ipsi  Ipsi | 3.02  4.02  3.09  2.69  4.53  3.27  2.77  2.39  3.01  4.39  6.47  3.10  3.04  2.99  2.91  3.18  2.57  5.10  6.43  3.21  6.93  4.20  4.57  6.44  4.63  4.42  2.68  5.82  5.22  3.17 |
