# Supplementary material for: Neural Substrates for the Motivational Regulation of Motor Recovery after Spinal-Cord Injury
Source: PLoS One. 2011 Sep 28;6(9):e24854. doi: 10.1371/journal.pone.0024854 (PMC3182173; doi:10.1371/journal.pone.0024854)
Supplement: Table S5 — Statistical analysis of correlation of the rCBF in the rACC with that in other brain regions during the intact, early, late stage of recovery and recovery stage. The same arrangement as Table S2. (DOCX) [file pone.0024854.s012.docx]

**Table S5**:

| Brain region | Laterality | t value |
| --- | --- | --- |
| Intact  Ca  M1  Cb  Cb  Early  OBF  Ca  VTA  Hip  IPS  Cb  Cb  V2    Late  OBF  PMd  Amygdala  Cb  Recovery  OBF  OBF  PreSMA  VSt  Pu  Ca  SMC  Insular  IPS  Cb  VTA  IPS  Cb  Cb | Ipsi  Ipsi  Contra  Ipsi  Ipsi  Contra  Contra  Contra  Ipsi  Contra  Contra  Contra  Ipsi  Contra  Ipsi  Ipsi  Ipsi  Contra  Mid  Contra  Contra  Contra  Contra  Contra  Contra  Ipsi  Contra  Ipsi  Ipsi  Contra | 3.02  4.02  3.09  2.69  4.12  3.74  3.14  3.40  3.90  3.18  2.65  3.54  3.51  3.06  2.83  3.43  3.57  3.26  4.97  3.57  2.69  3.41  2.65  3.38  3.91  4.25  2.92  3.53  3.45  4.21 |
