# Supplementary material for: Neural Substrates for the Motivational Regulation of Motor Recovery after Spinal-Cord Injury
Source: PLoS One. 2011 Sep 28;6(9):e24854. doi: 10.1371/journal.pone.0024854 (PMC3182173; doi:10.1371/journal.pone.0024854)
Supplement: Table S6 — Statistical analysis of correlation of the rCBF in the cACC with that in other brain regions during the intact, early, late stage of recovery and recovery stage. The same arrangement as Table S2. (DOCX) [file pone.0024854.s013.docx]

**Table S6**:

| Brain region | Laterality | t value |
| --- | --- | --- |
| Intact  OBF  OBF  rACC  Ca  PMd  M1  M1  Early  OBF ipsi  OBF contra  rACC mid  VSt contra  M1 ipsi  M1 contra  VTA contra  Cb mid  Cb contra  Cb Vermis mid  Late  M1 contra  SMA mid  M1 contra  Recovery  OBF contra  OBF ipsi  VSt contra  VSt ipsi  Pu contra  PMd contra  M1 contra  M1 ipsi  Insular contra  VTA bilateral  IPS contra  Cb ipsi  Cb Vermis mid  V1 contra | Ipsi  Ipsi  Ipsi  Contra  Contra  Contra  Ipsi  Ipsi  Contra  Mid  Contra  Ipsi  Contra  Contra  Mid  Contra  Mid  Contra  Mid  Contra  Contra  Ipsi  Contra  Ipsi  Contra  Contra  Contra  Ipsi  Contra  Bilateral  Contra  Ipsi  Mid  Contra | 3.23  4.22  3.34  3.86  5.47  2.71  2.61  3.55  2.93  4.86  3.01  3.93  3.10  2.75  3.52  4.45  4.14  2.90  3.98  3.47  4.74  3.81  3.55  3.28  2.91  3.08  6.25  4.68  4.68  3.55  5.57  3.44  4.43  3.86 |
