# Supplementary material for: Neural Substrates for the Motivational Regulation of Motor Recovery after Spinal-Cord Injury
Source: PLoS One. 2011 Sep 28;6(9):e24854. doi: 10.1371/journal.pone.0024854 (PMC3182173; doi:10.1371/journal.pone.0024854)
Supplement: Table S7 — Statistical analysis of correlation of the rCBF in the ip-PPTN with that in other brain regions during the intact, early, late stage of recovery and recovery stage. The same arrangement as Table S2. (DOCX) [file pone.0024854.s014.docx]

**Table S7**:

| Brain region | Laterality | t value |
| --- | --- | --- |
| Intact  OBF  46v  Amygdala  Cb  Cb  Cb  Early  Pu  SMA  S2/Insular  M1  Cb    Late  Cb    Recovery  OBF  OBF  VSt  VSt  PMv  M1  M1  Insular  AIP  IPS  Cb  Cb Vermis | Ipsi  Contra  Contra  Contra  Ipsi  Ipsi  Ipsi  Contra  Contra  Contra  Ipsi  Contra  Ipsi  Contra  Ipsi  Contra  Ipsi  Ipsi  Contra  Contra  Contra  Ipsi  Ipsi  Mid | 3.09  2.53  2.53  3.66  2.95  3.02  2.67  2.67  4.22  2.81  2.95  3.30  2.58  2.95  3.73  3.09  2.66  2.87  3.30  4.29  3.44  2.80  3.33  2.59 |
