# Supplementary material for: Neural Substrates for the Motivational Regulation of Motor Recovery after Spinal-Cord Injury
Source: PLoS One. 2011 Sep 28;6(9):e24854. doi: 10.1371/journal.pone.0024854 (PMC3182173; doi:10.1371/journal.pone.0024854)
Supplement: Text S1 — Supporting information for Methods. (DOCX) [file pone.0024854.s015.docx]

**Text S1**

**Detailed experimental procedure of PET scan**

High-resolution whole-brain MRI images were obtained using a conventional T1-weighted, fast-spin echo sequence (in-plane resolution, 0.80 × 0.80 mm; slice thickness, 0.81 mm) with a 3.0 Tesla MR imager. After taking an anatomical image of the brain using MRI, an acrylic head holder was attached to the skull. The head holder was used for the painless fixation of the monkey’s head during PET scanning. Thirty-one slices with a center-to-center distance of 3.6 mm were collected simultaneously by PET scanner in 3D mode. The transaxial resolution of the PET scanner was 2.6 mm at full-width-half-maximum (FWHM). Before the PET scan, each monkey was seated in a primate chair and transferred to the PET scanner without anesthesia. A 30 min transmission scan with a rotating ^68^Ge-^68^Ga pin source was obtained to evaluate the relative attenuation factor for image reconstruction. PET scans were performed in an upright sitting position in a scanner that was tilted at 80° from the vertical position to expand the view of the monkey. After the delivery of a bolus of [^15^O]H_2_O (~300 MBq in 1.5 ml followed by 1.0 ml of saline) via a cannula placed into the sural vein, the scan was initiated automatically when the radioactivity in the brain was greater than 30 kcps. The monkey was allowed to begin the behavioral task (20 trials) at about 10 s before the start of the PET scan. During the scan, the monkeys performed a series of reach-grip-retrieve-eat movements every 5 s. In addition, brain activation during the control task was recorded in separate sessions randomly mixed with the sessions of the precision grip task. Approximately 20 PET scans were performed per day with an inter-scan interval of about 15 min. PET experiments were conducted twice a week.

The cortical borderline and spatial coordination of [^15^O]H_2_O PET images was determined by the [^18^F]2-fluoro-2-deoxy-D-glucose ( [^18^F]FDG) PET scan conducted once in each subject by injecting a bolus of [^18^F] FDG (40 MBq/kg).

**FIGURE LEGENDS IN SUPPORTING DATA**

**Figure S1**. Strength of functional connectivity derived from correlations between the rCBF value in the co-VSt and that in other regions during the precision grip task were calculated. The data were obtained from 3 monkeys. Brain areas with a significant positive correlated rCBF (*P* < 0.01, uncorrected for multiple comparisons) are superimposed on a three-dimensional reconstruction of a template brain MRI of macaque monkeys that was made by our group. The significance level is given in terms of *t* values represented on a colored scale. (A), (B) (C) and (D) are results during the intact, early and late stages of recovery, and all recovery stages (including the data from both the early and late recovery stages), respectively. (a) to (g), coronal sections. (C) shows lateral views of the brain. Lines (d) to (f) in (C) indicate the levels of coronal sections of (a) to (g), respectively.

**Figure S2**. Strength of functional connectivity with the co-M1. The same arrangement as Figure S1.

**Figure S3**. Strength of functional connectivity with the co-OBF. The same arrangement as Figure S1.

**Figure S4**. Strength of functional connectivity with the rACC. The same arrangement as Figure S1.

**Figure S5**. Strength of functional connectivity with the cACC. The same arrangement as Figure S1.

**Figure S6**. Strength of functional connectivity with the ip-PPTN. The same arrangement as Figure S1.

**TABLE LEGENDS IN SUPPORTING INFORMATION**

**Table S1**: Statistical analysis of correlation of the rCBF between the co-VSt and the primary motor cortex at different stages before and after the spinal-cord injury. The values indicate Pearson’s correlation coefficients (r) and *p*-values. r and p-values correspond with each scatter plot in figure 2B in main text. “*n.s.*” indicates no significant. co-M1: contralateral M1, ip-M1: ipsilateral M1

**Table S2**: Statistical analysis of correlation of the rCBF in the co-VSt with that in other brain regions during the intact, early, late stage of recovery and recovery stage. The level of the coefficients was set at *P* < 0.01 (t=2.38 for the intact, early, late stage of recovery, t=2.35 for recovery stages). *t*-values at the center of individual masses of activation are indicated.

**Table S3**: Statistical analysis of correlation of the rCBF in the co-M1 with that in other brain regions during the intact, early, late stage of recovery and recovery stage. The same arrangement as Table S2.

**Table S4**: Statistical analysis of correlation of the rCBF in the co-OBF with that in other brain regions during the intact, early, late stage of recovery and recovery stage. The same arrangement as Table S2.

**Table S5**: Statistical analysis of correlation of the rCBF in the rACC with that in other brain regions during the intact, early, late stage of recovery and recovery stage. The same arrangement as Table S2.

.

**Table S6**: Statistical analysis of correlation of the rCBF in the cACC with that in other brain regions during the intact, early, late stage of recovery and recovery stage. The same arrangement as Table S2.

**Table S7**: Statistical analysis of correlation of the rCBF in the ip-PPTN with that in other brain regions during the intact, early, late stage of recovery and recovery stage. The same arrangement as Table S2.

**Abbreviations for table**: AIP, Anterior intraparietal cortex; rACC, rostral part pf the anterior cingulate cortex; cACC, caudal part pf the anterior cingulate cortex; Ca, Caudate; Cb, Cerebellum; contra (co-), contralateral hemisphere to the spinal-cord injury; Hip, Hippocampus; IPS, intra parietal sulcus; ipsi (ip-), ipsilateral hemisphere to the spinal-cord injury; M1, primary motor cortex; VSt, ventral striatum; OBF, orbitofrontal cortex; PMd, dorsal premotor cortex; PMv, ventral premotor cortex; Pu, putamen; PCC, posterior cingulate cortex; PPTN, pedunculopontine tegmental nucleus; PreSMA, pre supplemental motor area; PN, pontine nucleus; SMA, supplemental motor area; SMC, sensory motor cortex; S1, primary sensory cortex; S2, secondary sensory cortex; TPO, temporal parieto-occipital area; V, visual cortex; VIP, ventral intraparietal area; VTA, ventral tegmental area; 46, area 46; 46v, ventral part of area 46.
